# Influence of Clinical Decontamination Techniques on the Surface Characteristics of SLA Titanium Implant

**DOI:** 10.3390/nano12244481

**Published:** 2022-12-18

**Authors:** Meltem Bayrak, Necla Asli Kocak-Oztug, Karan Gulati, Serdar Cintan, Emine Cifcibasi

**Affiliations:** 1 Faculty of Dentistry, Department of Periodontology, Istanbul University, Istanbul 34116, Turkey; 2 Institute of Graduate Studies in Health Sciences, Department of Periodontology, Istanbul University, Istanbul 34126, Turkey; 3 School of Dentistry, The University of Queensland, Herston, QLD 4006, Australia

**Keywords:** peri-implantitis, decontamination, surface roughness, titanium, hydrophilicity, SLA

## Abstract

The study aims: 1. To perform diode laser, titanium (Ti) brush, and Ti curette treatment on sandblasted and acid-etched (SLA) Ti surfaces, with/without H_2_O_2_ and CHX, 2. To investigate the influence of decontamination techniques on implant surface topography and hydrophilicity. Diode laser, Ti brush, and Ti curette treatments were performed on the Grade 4 Ti discs, with/without treatment with 3% H_2_O_2_ solution or 0.2% CHX. Surface characteristics were investigated via SEM, optical profilometry, and water contact angle meter. SEM findings revealed flat and scratched areas when treated with Ti curette and Ti brush. For diode laser, SEM showed melting in specific areas. Ra and Rt values were lower in all test groups than in the control group (*p* < 0.05). The adjunctive chemical treatment showed negligible effects in SEM images and surface roughness measurements compared to laser and mechanical treatment-only groups. H_2_O_2_ treatment resulted in enhanced hydrophilicity in either treatment modalities with a significant difference compared to the negative control group (*p* < 0.05). In all test groups, the hydrophilicity was enhanced compared to the negative control group (*p* < 0.05). Diode laser treatment had the least disruptive effect on the Ti surface characteristics. The use of other mechanical methods caused significant alterations in the surface roughness.

## 1. Introduction

Peri-implantitis is a plaque-associated pathological disease related to bleeding, suppuration, and bone loss affecting implant-surrounding soft and hard tissues [1,2]. The etiology of peri-implantitis contains many determinants, such as the implant design, surface topography/roughness, surrounding tissue condition and the surgeon’s experience [3,4]. In any case, plaque accumulation on the implant surface plays a significant role during peri-implant disease progression. Although plaque accumulation is the primary etiologic factor of both periodontitis and peri-implantitis, the treatment of peri-implantitis is challenging, due to the varied implant topographical characteristics [4,5,6]. Therefore, surface decontamination is vital for the treatment of peri-implantitis. Several implant decontamination techniques have been reported to remove biofilms on the implant surface, including surgical and non-surgical methods. Non-surgical therapy involves mechanical treatments such as metal, plastic and titanium (Ti) curettes, Ti and nylon brushes, and ultrasonic scalers. Additionally, non-surgical therapy involves chemical treatments, including chlorhexidine digluconate (CHX), citric acid, hydrogen peroxide (H_2_O_2_), and saline [4,7,8,9]. It is noteworthy that plastic curette applications can influence implant surfaces that can cause disruptions in cell adhesion and the bone healing process. This could be associated to the accumulation of the plastic curette residues that may impair cell attachment and proliferation. Chemical treatments may also affect the physicochemical characteristics of implant surfaces, which can alter implant bioactivity. Moreover, absorbed CHX by Ti surfaces could alter cellular re-attachment [4,7,8,9]. The current information supports that mechanical and chemical treatments can cause implant surface alterations. However, the impact of these changes on clinical outcomes remains inferiorly understood and explored [10].

Recently, low-power and high-power lasers have been utilized as an alternative non-surgical decontamination technique for dental implants [8]. Low power lasers such as diode laser, are commonly used in dental clinics, due to their ability to achieve hemostasis, and bactericidal action. In addition, low-power lasers are easily accessible and cheaper than high-power lasers. In the literature, various treatment protocols are proposed for the use of lasers for implant surface decontamination [11,12]. However, laser decontamination of implant surfaces to combat peri-implantitis is poorly optimized [12,13].

Decontamination of the Ti implant surface is challenging, and is attributed to the specific bioactive surface topography that can offer shelter for bacterial adhesion and proliferation [14,15]. An ideal surface decontamination technique would enable the effective removal of bacterial residues and the preservation of the topographic modifications of the implant [16]. Clinically, decontamination methods are considered safe in treating peri-implantitis; however, its influence on implant surface features remains inadequately explored [17,18].

It is well established that the surface topography/roughness of the Ti implants influences osseointegration [14]. Cellular functions such as proliferation, differentiation, and adhesion are influenced by chemical composition, roughness, and wettability of the implant, thus affecting osseointegration and the long-term success of implant treatment [19].

Albrektsson and Wennerberg demonstrated that higher surface roughness accelerates bone formation [14]. According to studies of Berglundh et al. dental implant surfaces were modified to be moderately rough [20]. Alternatively, hydrophilicity also strongly influences implant performance [19,21]. Various strategies have been employed to improve implant surface hydrophilicity and bioactivity, via physical, chemical and biological modifications, as described elsewhere [22]. Within these techniques, sandblasted and acid-etched surfaces (SLA) have exhibited superior implant–cell interactions and become a standard surface preference for most clinically-utilized dental implants [23,24]. This surface modification not only augments bone healing activity but also enables enhanced primary stability at the implant–bone interface. While surface roughness promotes bioactivity and integration, it also enhances plaque accumulation. Hence, to ensure the long-term success of roughened/modified dental implants, especially in compromised patient conditions, effective implant decontamination strategies are needed that do not negatively influence implant bioactivity by altering surface topography [23,24,25].

According to recent studies, due to the variations of the proposed decontamination techniques, there is a lack of reliable evidence to indicate the most effective intervention for the treatment of peri-implantitis [16,26]. Further examination of the possible detrimental effects of various decontamination methods on implant surfaces will assist clinicians in choosing the most reliable technique. For the above-mentioned research gap, this study aimed to evaluate the changes in surface topography, roughness and wettability of Ti SLA discs after performing mechanical decontamination methods (Ti curettes, Ti brushes), and laser (diode) irradiation methods, in combination with/without chemical agents (CHX, H_2_O_2_).

## 2. Materials and Methods

### 2.1. Titanium Disc Preparation

In this study, a total of 50 SLA surface grade 4 Ti discs (Trias-ixx2 Implant Systems, Servo Dental GmbH), with a diameter of 10 mm and 1 mm in thickness and an arithmetic mean deviation of the roughness profile (Ra) = 2.25 μm, were used. Five Ti discs were used for each group. Ti discs were sequentially sonicated with acetone, absolute ethanol, deionized water for 15 min, rinsed with distilled water and autoclaved for 15 min at 121 °C. 

### 2.2. Implant Surface Treatment

In our study, 1 control group and 9 test groups were investigated. No process was applied to the control group after sterilization. The determined treatment procedures were applied to test groups [18]. The procedures were performed by the same researcher (M.B.) for standardization purposes. Test groups are detailed in Table 1.

### 2.3. Surface Characterization of Titanium Discs

#### 2.3.1. Scanning Electron Microscopy (SEM)

The topographic surface alteration analysis of the specimens was performed using SEM (FEI™ VERSA 3DLOVAC, Hillsboro, OR, ABD). The Ti discs were imaged by SEM at 20 kV, and the magnification used in the images were 2000x. After the treatment procedures, 3 areas were randomly selected from each disc for SEM to observe the topography of Ti discs.

#### 2.3.2. Surface Roughness Measurement

The surface roughness of each Ti disc was measured using a surface profilometer (Veeco Instruments Inc., Plainview, NY, USA, ABD). In addition to the arithmetic mean deviation of the roughness profile (Ra), other roughness parameters such as the maximum height of profile (Rt) and root mean square deviation of the roughness profile (Rq) were also evaluated. In each case, the measurement was performed with a 0.25 mm cut-off and over an assessment length of 1.25 mm. Each specimen was measured at 0.5 mm intervals lengthwise and widthwise, from which the average for each specimen was calculated.

#### 2.3.3. Contact Angle Measurement

The static contact angles of each Ti disc were measured to quantify surface hydrophilicity using the sessile drop method with water at room temperature (25 ± 2 °C) using the KSV CAM 200 instrument. Deionized water was used as the wetting liquid with a drop volume of 2 μL. After the water drop was casted on the surface, images were captured, and contact angles were calculated using the tangent method. Five recurrent analyses were performed for each sample. For the control group, the measurements were performed twice, first on the dry discs (negative-dry control group) and second after soaking the discs in sterile saline solution for 60 s (positive-wet control group) to gain similar wetness to the test groups.

### 2.4. Statistical Analysis

The total sample size found using the g-power program with an effect size of 0.72, a power of 90%, and a margin of error of 0.5 was n = 50. The number of samples for the ANOVA test method was determined by considering 10 independent groups based on the calculation resulting in 5 discs in each group. The NCSS (Number Cruncher Statistical System) 2007 (Kaysville, UT, USA) program was used for statistical analysis. Descriptive statistical methods (mean, standard deviation, median, frequency, percentage, minimum and maximum) were used while evaluating the study data. The Kruskal-Wallis test and Dunn-Bonferroni test were used to compare groups of more than two quantitative variables that did not show normal distribution. The statistical significance was accepted as *p <* 0.05.

## 3. Results

### 3.1. Influence of Decontamination Treatment on Surface Topography

**Figure 1:**
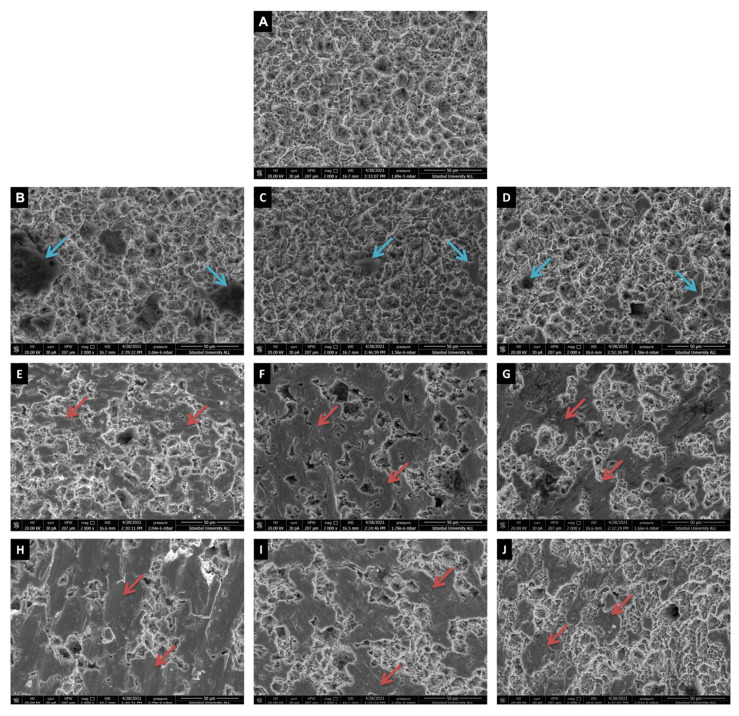
SEM images of SLA Ti discs. (**A**) Control group, (**B**) Laser, blue arrows point to melting areas, (**C**) Laser + H_2_O_2_, (**D**) Laser + CHX, (**E**) Ti-brush, red arrows point to smooth-flattening areas, (**F**) Ti-brush + H_2_O_2_, (**G**) Ti-brush + CHX, (**H**) Ti-curette, (**I**) Ti-curette + H_2_O_2_ and (**J**) Ti-curette + CHX. Images of each group demonstrate 2000x magnification.

According to SEM analysis, the control (untreated) implant showed a rough and irregular honeycomb appearance, which is characteristic of SLA surfaces (Figure 1A) [29]. In the laser treatment group, slight topographical changes (e.g., flattening) due to melting and hence smoother surface areas were observed as compared to the control group (Figure 1A–D). Laser treatment combined with both chemical agents showed similar surface alterations compared to laser treatment alone (Figure 1B–D). Similar microtopographic alterations were observed on the surface of two different mechanical therapy methods (Figure 1E–J). Both Ti brush and Ti curette treatments showed scratches and flattened areas at 2000x (Figure 1E–J). The appearance of the honeycomb was observed to turn into solid flat areas. When mechanical and chemical treatments were applied in combination, no visual difference was observed compared to the mechanical treatment only (Figure 1E–J).

### 3.2. Surface Roughness Changes with Implant Decontamination

**Figure 2:**
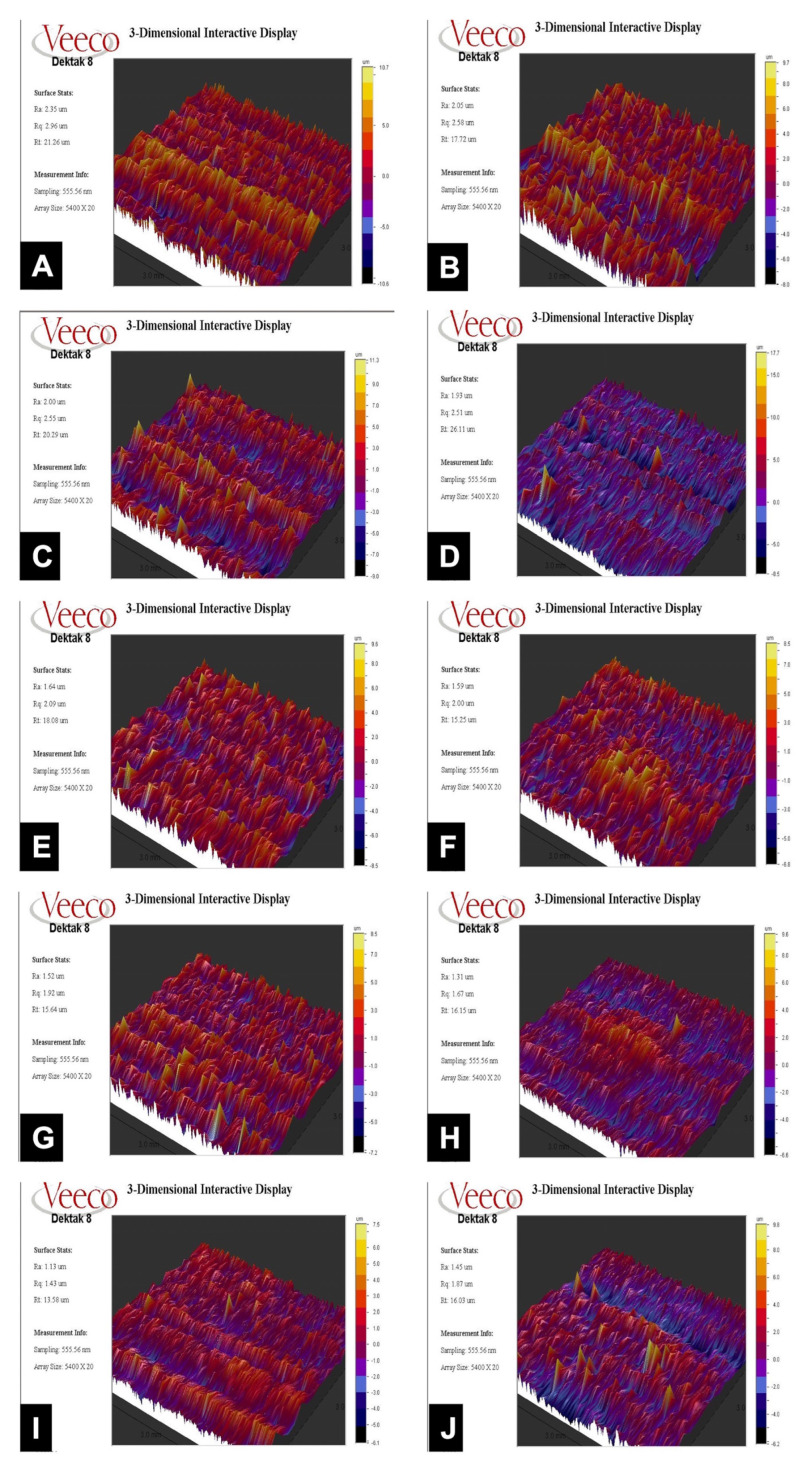
Optic profilometry analysis of various Ti implant surfaces: (**A**) Control untreated, (**B**) Laser, (**C**) Laser + H_2_O_2_, (**D**) Laser + CHX, (**E**) Ti brush, (**F**) Ti brush + H_2_O_2_, (**G**) Ti brush + CHX, (**H**) Ti curette, (**I**) Ti curette + H_2_O_2_, and (**J**) Ti curette + CHX.

Profilometry analysis results are shown in Figure 2A–J. The surface roughness values Ra, Rq and Rt are presented in Table 2.

In the initial statistical analysis, a significant difference among the Ra measurements of the groups was found (*p* < 0.01) (Table 2). Subsequently, pairwise comparisons between the groups were performed. According to the secondary evaluation, the Ra measurements in the Ti-curette + H_2_O_2_ group were significantly lower than those in the control group and laser group (*p* < 0.01). Likewise, the Ra measurements in the Ti-curette group were significantly lower than those in the control group and laser group (*p* < 0.05), and the Ra measurements in the Ti curette + CHX group were significantly lower than those in the control group (*p* < 0.05) (Table S1).

In the initial statistical analysis, a significant difference was found among the Rq measurements of the groups (*p* < 0.01) (Table 2). Subsequently, pairwise comparisons between the groups were performed. According to the secondary evaluation, the Rq measurements of the Ti curette + H_2_O_2_ group were significantly lower than those in the control group and laser group (*p* < 0.05). Likewise, Rq measurements in the Ti curette group were significantly lower than those in the control group and laser group (*p* < 0.05) (Table S2).

In the initial statistical analysis, a significant difference was found among the Rt measurements of the groups (*p* < 0.01) (Table 2). Subsequently, pairwise comparisons between the groups were performed. According to the secondary evaluation, the Rt measurements of the Ti curette + H_2_O_2_ group were significantly lower than those in the control group, laser + H_2_O_2_ group, laser + CHX group and laser group (*p* < 0.05). In addition, the Rt measurements of the Ti Brush group were significantly lower than those in the laser + H_2_O_2_ group, laser + CHX group and laser group (*p* < 0.01). The values in the Ti Brush + CHX group were significantly lower than those in the laser, laser + H_2_O_2_ and laser + CHX groups (*p* < 0.01) (Table S3).

### 3.3. Surface Decontamination Alters Implant Wettability

**Figure 3:**
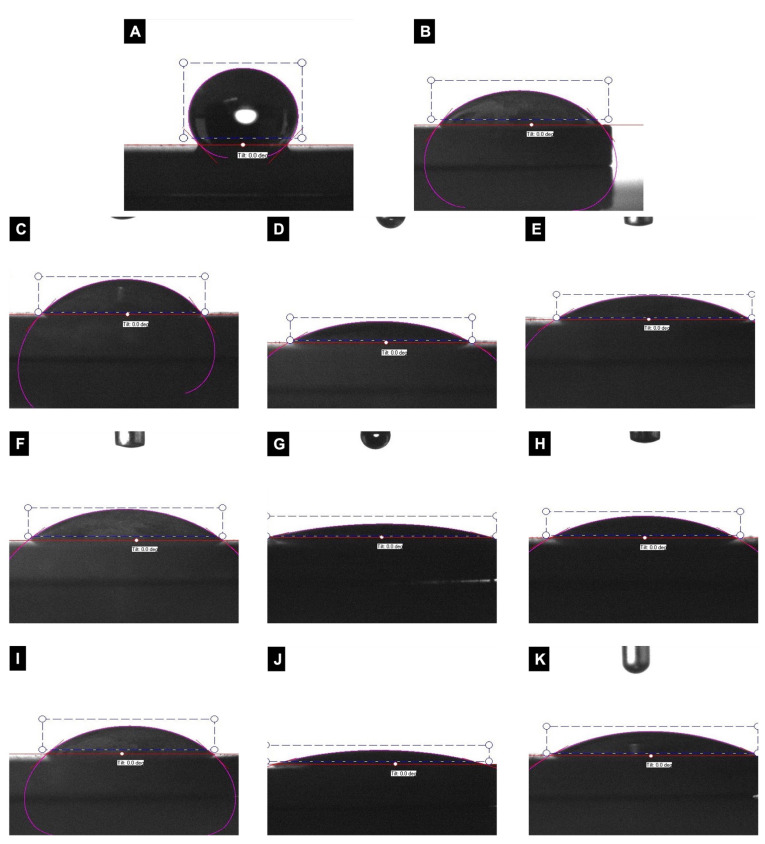
Representative images of contact angle measurement for various treated implant surfaces: (**A**) Negative-dry control group, (**B**) Positive-wet control group, (**C**) Laser, (**D**) Laser + H_2_O_2,_ (**E**) Laser + CHX, (**F**) Ti brush, (**G**) Ti brush + H_2_O_2_, (**H**) Ti brush + CHX, (**I**) Ti curette, (**J**) Ti curette + H_2_O_2_ and (**K**) Ti curette + CHX.

In the initial statistical analysis, a significant difference between the contact angles of the groups was found (*p* < 0.01) (Table 3). Subsequently, pairwise comparisons between the groups were performed. According to the secondary evaluation, the contact angles in the negative control group were significantly higher than those in the Laser + H_2_O_2_, Ti Brush + H_2_O_2_, Ti Brush + CHX, Ti curette + H_2_O_2_ and Ti curette + CHX groups (*p* < 0.01). Likewise, the contact angles of those in the laser group were significantly higher than those in the Ti Brush + H_2_O_2_ and Ti curette + H_2_O_2_ groups (*p* < 0.01). The contact angles in the Ti Brush group were significantly higher than in the Ti Brush + H_2_O_2_ and Ti curette + H_2_O_2_ groups (*p* < 0.05). Contact angles of those in the Ti Brush + H_2_O_2_ group were significantly lower than in the Ti curette group (*p* < 0.05). Contact angles in the Ti curette + H_2_O_2_ group were significantly lower than in the Ti curette + CHX group (*p* < 0.01) (Table S4 and Figure 3).

## 4. Discussion

Due to frequent implant usage in dentistry, gradual failures related to peri-implantitis have been recorded [26]. Consequently, the treatment of peri-implantitis has become a consistent challenge for daily clinical practice [30]. The elimination of biofilms is the central goal of peri-implantitis treatment, and peri-implant biofilm removal is more complex than periodontal biofilm removal due the morphology and roughness of the implant surface. As reported by Berglundh et al., the decontamination of rough surfaces, such as SLA surfaces, is more complicated than machined surfaces, and hence further optimizations are required to treat peri-implantitis in SLA surfaces [20].

Non-surgical techniques to treat peri-implantitis mainly include the administration of antibiotics or antiseptics, and the use of Ti curette and laser debridement. On the other hand, surgical procedures include additional reconstructive and regenerative surgery after debridement [31]. Although numerous peri-implantitis treatment procedures are available, none of the techniques can provide adequate biofilm removal without causing alterations to the implant surface. In addition, there is no clear guideline for the techniques that have been recommended [31,32,33]. Further, when clinicians try to eliminate the biofilm during peri-implantitis treatment, they may not visualize the implant surface completely due to various factors, including implant position, flap design and anatomical structure. These challenges can cause them to continue the decontamination treatment even after biofilm removal, damaging the implant surface. In recent research, repeated mechanical and laser treatments for non-surgical peri-implantitis treatment are also on the horizon, which might increase the risk of surface alteration [24,34]. In vitro studies, including ours, focus on the knowledge necessary to assist the end user in treating peri-implantitis. In this regard, we aimed to investigate the effects of commonly used non-surgical peri-implantitis treatment methods on SLA Ti discs by evaluating the changes in surface topography, roughness and wettability (Table 1).

Our findings demonstrated that when the diode laser was applied at λ = 808 ± 10 nm under water irrigation for 60 s, there were minor effects on the morphology of SLA Ti surfaces (Figure 1). Regarding hydrophilicity, the laser treatment groups’ values were higher than the positive control, and lower than the negative control (Table 3). Furthermore, the surface roughness was slightly reduced (2.2 μm vs. 2.08 μm) (Table 2 and Table S1). SEM analyses of the laser groups also revealed that the treated surfaces displayed a qualitatively similar surface morphology to the control group (Figure 1). These findings demonstrate that when a diode laser with λ 808 and 1 W power energy was used for 60 s, the surface integrity was preserved on Ti SLA surfaces, except for minor changes (melting and flattening in certain areas not covering all surfaces) in specific regions (Figure 1). Regarding the diode laser, the findings of this study are partially consistent with the literature [17,35]. In our study, SEM images of laser groups revealed minor melting regions. On the other hand, Castro et al. reported no significant Ti surface damage when treated with a diode laser at a relatively higher power (15 W) [36]. This may rely on the treatment duration used in that study (60 s) for the entire implant surface. Different laser systems (including the erbium-doped yttrium–aluminium–garnet (Er:YAG) laser, neodymium-doped yttrium–aluminium–garnet (Nd:YAG) laser and carbon dioxide (CO_2_) laser have been developed and performed on various implant surfaces [5,37,38,39]. Nevertheless, it can be quite challenging for a practitioner to select the best laser settings for a particular implant surface to treat peri-implant disease. Lower device costs dictate the rise in diode laser usage by clinicians. However, there remains a debate over how to prevent tissue heat injury [35]. The prolonged treatment time on the relatively smaller surface may contribute to surface warming, as observed in our study, and in others [40]. The results of a recent review to demonstrate whether CO_2_, Er:YAG and diode laser therapy is effective for treating peri-implantitis found that the heterogeneity and a limited number of included publications made it impossible to conclude which laser therapy was superior [41]. Giannelli et al. stated that the 808-nm diode laser could effectively reduce the *S. aureus* biofilm and detoxify LPS on the Ti surface [11]. Notably, in both the continuous and pulsed wave modes, the irradiation of the biofilm-coated discs for 1 min was able to reduce the number of colony-forming units up to 99 and 94 percent, respectively [11]. Furthermore, in our study, an additional application of CHX or H_2_O_2_ chemical treatment to the mechanical and laser treatment did not result in visible changes on the implant surface.

In our study, during SEM evaluation, the surfaces treated with the Ti curette and Ti brush revealed noticeable changes in the surface microstructure, such as flattening, scratches and deformation of the honeycomb appearance. This change in surface topography can alter the implant’s bioactivity performance. Some Ti particle residues were also observed in the SEM images under 2000x in the Ti Brush group (Figure 1). In parallel with our findings, Lollobrigida et al. found residues on the Ti surfaces after the treatment with a Ti brush [40]. Although the outcomes in the literature are not encouraging for preserving surface micro-topography, Ti or carbon fibre curettes are frequently used in mechanical debridement to treat peri-implantitis [40]. After the mechanical treatment of peri-implantitis, the presence of grooves, scratches, and undesirable surface modifications caused by instrumentation can cause plaque and calculus formation more easily.

The dental implant surface topography is a fundamental element of the osseointegration process [42,43]. The amount and formation of dental plaque are also impacted by the implant surface roughness [43]. Nevertheless, there is no adequate data demonstrating a relationship between implant surface roughness characteristics and the structure of the developing biofilm [44]. Optical profilometry is a well-established analyzing method for dental implant surface roughness [30]. With the help of this technique, three-dimensional surface images can be documented, and topographic properties can be evaluated. The arithmetic mean of the elevation profile Ra, the mean square elevation profile Rq and the maximum height of the profile Rt are typically used to define micro surface geometries [45]. According to the results of our study, Ra values decreased in the Ti curette and the Ti brush groups. Ra value did not make a significant difference in the laser group (Table 2 and Table S1). In contrast with our findings, Park et al. stated that after Ti Brush treatment to the SLA and machined surface, the discs did not produce significant changes in the roughness parameters, including Sa (extension of Ra) and Sz (extension of Rz) [46]. The differences between the studies may be attributed to the change in rotation speed and time. In our study, 920 rpm for 60 s was used, compared to 300 rpm for 40 s in the other study.

Surface roughness and topography play a significant role in predicting the success and survival of an implant [14]. Increased blood cell contact with the Ti surface improves platelet adhesion and osteogenic chemotaxis on rough Ti surfaces, resulting in improved direct osteogenesis and bone–implant contact [13,14,47]. Enhanced phenotypic expression and more differentiated osteoblast phenotypes may result from the increased interaction between osteoblasts and implant surfaces [13]. It was well established that bone-to-implant contact significantly increases with larger surface roughness, even though the ideal surface roughness is still debatable [20,47]. On the other hand, detrimental impact of surface roughness must also be considered. High surface roughness may stimulate early plaque formation via exposure to the oral cavity in peri-implantitis [20,23]. Implant surfaces are categorized into four different groups based on tridimensional roughness (Sa values): smooth (Sa 0.5 m), minimally rough (Sa = 0.5–1.0), moderately rough (Sa = 1.0–2.0 m) and highly rough (Sa > 2.0 m) [48,49]. Bidimensional roughness media values (Ra) less than or equal to 1 m are typically considered smooth, whereas those more than 1 m are rough [48,49]. In our study, the mean Ra value of the control group was 2.25 μm, which is highly rough (Table 2). After laser treatment, the value decreased to 2.08 μm (nearly moderately rough), and after Ti brush and Ti curette treatment, surface roughness values reduced to 1.62 μm and 1.22 μm, respectively (Table 2). Upon application of H_2_O_2_ or CHX to the test groups, no significant changes in the surface roughness values were observed (Table 2 and Table S1–S3). Similar to the results of our study, Homayouni et al. reported that using 3% H_2_O_2_ as an adjunctive agent did not alter the surface roughness as compared to the control group [17]. As expected in our study, Rq values revealed similar results to Ra values in group comparisons (Tables S1 and S2). On the other hand, the Rt value of the laser-treated group was higher than that of the control group, while the values of the other test groups showed similar results with the Ra and Rq values (Table S1–S3).

Regarding dental implant applications, material surface characteristics, including wettability, have drawn much attention in recent years [21,49]. The contact angle between the water droplet interface and the horizontal surface can be used to estimate the hydrophilicity of the implant surface. Surfaces are termed hydrophilic when the contact angle is less than 90 degrees, and hydrophobic when the contact angle is more than 90 degrees. The wettability of implant surface impacts the interaction with the biological environment [21]. Highly hydrophilic surfaces interact with biological features better than hydrophobic surfaces. Thus, improving the wettability of implant surfaces is a significant modification. According to the general theory, increasing wettability improves biocompatibility, fostering interactions between an implant surface and the biological environment and enabling the activation of cellular activity [19,21]. Even with current developments to enhance the surface hydrophilicity, most implant surfaces in the market are in the hydrophobic range [50,51]. Additionally, wettability encourages protein adsorption, cellular adhesion and proliferation [16]. The present study demonstrated that contact angle measurements were higher after laser treatment than in the positive control group (Table 3). Further, water contact angles in mechanical treatment groups were also higher than those of the positive controls (Table 3 and Figure 3). Regarding chemical treatments, contact angles were lower than those in the control groups and test groups with no adjunctive chemical agents, which could be caused by CHX and H_2_O_2_ absorption of SLA surfaces, as previously reported by Kozlovsky et al. and Boonstra et al. (Table 3 and Table S4) [52,53].

In our study, the negative control group (untreated/dry) contact angle measurements were higher than the positive control (wet) and test groups (Table 3 and Table S4). Positive group discs were soaked in saline solution to mimic the effects of cooling with saline during treatment. To the best of our knowledge, this is the first study comparing positive and negative controls with different treatment groups in a similar setting. As parallel to our findings, Matthens et al. reported that contact angle measurements after mechanical treatment (curette, water jet and cold atmospheric pressure plasma treatment) were reduced compared to the untreated positive control group [54]. Moreover, Lee et al. also observed reduced hydrophilicity (high water contact angle) after mechanical treatment [28].

It is noteworthy that we used Ti SLA discs instead of dental implants in our study, which could be a drawback. Although Ti SLA discs have a microstructure similar to clinically utilized dental implant surfaces, testing implant treatment with screw threads is challenging. Nevertheless, it has been demonstrated that the methodology employed in this study is effective for comparing various treatment techniques by several studies.

Studies with similar aims have generally focused on the biofilm removal efficacy of peri-implantitis treatment methods. In the current study, we bypassed the biofilm growth while investigating the influence of decontamination methods on implant surface to minimize any shielding effect caused by the biofilm. Considering that decontamination is a routine procedure in clinical dentistry, and with varied treatment modalities and conditions (such as wavelength, duration, intensity or area of exposure for laser), level of expertise and extent of peri-implantitis, investigating surface characteristics of a clean implant surface allows for adequate influence approximation.

## 5. Conclusions

Dental implant surface topography and bioactivity performance can be influenced by decontamination techniques used to treat peri-implantitis. In this study, we systematically investigated the influence of common peri-implantitis treatment strategies, including laser, Ti brush and Ti curette use, with/without chemical treatment with H_2_O_2_ and CHX. Our study revealed that diode laser treatment had a minimum influence on SLA surface characteristics compared to mechanical decontamination treatments. Understanding the precise effect of surface decontamination on an implant surface characteristic can enable effective decontamination, without negatively impacting the implant’s bioactivity and performance. Future research requires additional systematic analyses of commonly used decontamination techniques to form clinical guidelines for various peri-implant disease treatments.

## Figures and Tables

**Table 1:** Summary of various decontamination treatments investigated on SLA Ti discs.

Group Name	Treatment	Details
Control	No treatment	Sterilized as-received SLA (Sandblasted-acid-etched) surface grade 4 Ti discs.
Group 1	Diode laser	Diode laser (Biolase, California, USA) was applied to Ti discs at a wavelength of λ = 808 ± 10 nm, 1 W (power) under continuous mode for 60 s. The laser tip was kept at a distance of 2–5 mm from the discs to prevent overheating, and saline cooling was applied to the entire surface during treatment [11,12].
Group 2	Diode laser + 3% H_2_O_2_	After laser treatment, the discs were immersed in 3% H_2_O_2_ solution for 60 s [17].
Group 3	Diode Laser + 0.2% CHX	After laser treatment, the discs were immersed in 0.2% CHX solution for 60 s [12].
Group 4	Ti brush	Ti brush (Ti-Brush, Straumann, Basel, Switzerland) was applied to Ti discs at 920 rpm under saline irrigation for 60 s. A new brush was used for each disc [27].
Group 5	Ti Brush + 3% H_2_O_2_	After Ti brush treatment, the discs were immersed in 3% H_2_O_2_ solution for 60 s.
Group 6	Ti Brush + 0.2% CHX	After Ti brush treatment, the discs were immersed in 0.2% CHX solution for 60 s.
Group 7	Ti curette	Ti discs were instrumented with a Ti curette (LM-ErgoMix™, Planmeca Group, Pargas, Finland) for 60 s in one direction at an angle of 45 degrees, and saline irrigation was applied to prevent surface heating [28]
Group 8	Ti Curette + 3% H_2_O_2_	After Ti-curette instrumentation, the discs were immersed in 3% H_2_O_2_ solution for 60 s.
Group 9	Ti Curette + 0.2% CHX	After Ti-curette instrumentation, the discs were immersed in 0.2% CHX solution for 60 s.

**Table 2:** Variation and initial statistical analysis of surface roughness values of Ti post decontamination treatment (values in μm). ^a^ Kruskal Wallis Test ** *p* < 0.01.

Groups		Ra	Rq	Rt
Control	Mean ± SD	2.25 ± 0.14	2.84 ± 0.17	24.39 ± 5.87
	Median (Min–Max)	2.3 (2.1–2.4)	3 (2.6–3)	22.1 (17.7–31.9)
Laser	Mean ± SD	2.08 ± 0.14	2.75 ± 0.31	32.23 ± 7.57
	Median (Min–Max)	2.1 (1.9–2.3)	2.7 (2.5–3.3)	31.3 (26.1–44.7)
Laser + H_2_O_2_	Mean ± SD	1.91 ± 0.32	2.51 ± 0.44	28.47 ± 6.36
	Median (Min–Max)	2 (1.5–2.4)	2.6 (1.9–3.1)	29.6 (18.4–35.7)
Laser + CHX	Mean ± SD	1.92 ± 0.10	2.53 ± 0.15	30.69 ± 8.57
	Median (Min–Max)	1.9 (1.8–2)	2.6 (2.3–2.7)	34.4 (20.3–38.4)
Ti Brush	Mean ± SD	1.62 ± 0.05	2.06 ± 0.09	16.68 ± 1.69
	Median (Min–Max)	1.6 (1.6–1.7)	2.1 (1.9–2.1)	17.5 (13.8–18.1)
Ti Brush + H_2_O_2_	Mean ± SD	1.68 ± 0.12	2.22 ± 0.25	20.27 ± 5.49
	Median (Min–Max)	1.7 (1.5–1.8)	2.3 (1.9–2.5)	18.6 (15.3–29.6)
Ti Brush + CHX	Mean ± SD	1.47 ± 0.06	1.87 ± 0.09	17.84 ± 4.40
	Median (Min–Max)	1.5 (1.4–1.5)	1.8 (1.8–2)	16.6 (13.4–25)
Ti Curette	Mean ± SD	1.22 ± 0.18	1.58 ± 0.24	18.13 ± 4.95
	Median (Min–Max)	1.2 (1–1.5)	1.6 (1.4–1.9)	16.2 (13.6–24)
Ti Curette + H_2_O_2_	Mean ± SD	1.14 ± 0.17	1.51 ± 0.29	15.99 ± 2.88
	Median (Min–Max)	1.1 (0.9–1.3)	1.4 (1.2–1.9)	14.5 (13.6–19.3)
Ti Curette+ CHX	Mean ± SD	1.42 ± 0.21	1.85 ± 0.25	20.75 ± 3.62
	Median (Min–Max)	1.4 (1.2–1.7)	1.9 (1.5–2.2)	22.6 (16–23.7)
	*p*	^a^ 0.001 **	^a^ 0.001 **	^a^ 0.001 **

**Table 3:** Contact angle measurements and initial statistical analysis of groups (values in degrees). ^a^ Kruskal Wallis Test ** *p* < 0.01.

		Contact Angle		*p*
		Mean ± SD	Median (Min–Max)	
**Groups**	Negative control	129.20 ± 10.03	131 (112–138)	^a^ 0.001 **
	Positive control	41.40 ± 8.91	37 (33–52)	
	1. Laser	53.40 ± 8.20	50 (44–63)	
	2. Laser + H_2_O_2_	23.80 ± 2.86	24 (20–27)	
	3. Laser + CHX	36.20 ± 4.66	35 (31–42)	
	4. Ti Brush	45.80 ± 3.70	46 (41–51)	
	5. Ti Brush+ H_2_O_2_	10.40 ± 2.97	10 (6–14)	
	6. Ti Brush + CHX	24.40 ± 4.16	25 (19–30)	
	7. Ti Curette	46.80 ± 4.97	46 (40–53)	
	8. Ti Curette + H_2_O_2_	12.60 ± 3.05	12 (9–17)	
	9. Ti Curette + CHX	21.40 ± 5.03	20 (17–30)	

## Data Availability

Data are contained within the article.

## Supplementary Materials

The following supporting information can be downloaded at: https://www.mdpi.com/article/10.3390/nano12244481/s1, Table S1: Pairwise comparison of Ra between groups via post hoc analysis. Dunn Bonferroni Test * *p* <0.05, ** *p* < 0.01; Table S2. Pairwise comparison of Rq between groups via post hoc analysis. Dunn Bonferroni Test * *p* < 0.05 ** *p* < 0.01; Table S3. Pairwise comparison of Rt between groups via post hoc analysis. Dunn Bonferroni Test * *p* < 0.05 ** *p* < 0.01; Table S4. Pairwise comparison of contact angle parameters between groups via post hoc analysis. Dunn Bonferroni Test * *p* < 0.05 ** *p* < 0.01.
