# Supplementary material for: Influence of Clinical Decontamination Techniques on the Surface Characteristics of SLA Titanium Implant
Source: Nanomaterials (Basel). 2022 Dec 18;12(24):4481. doi: 10.3390/nano12244481 (PMC9784882; doi:10.3390/nano12244481)
Supplement: Supplementary file 1 [file nanomaterials-12-04481-s001.zip › nanomaterials-2019404-supplementary.pdf]

Table S1. Pairwise comparison of Ra between groups via post hoc analysis. Dunn Bonferroni Test *$p<0.05$, **$p<0.01$

Ra	Control	Laser	Laser + H_2O_2	Laser + CHX	Ti Brush	Ti Brush+ H_2O_2	Ti Brush + CHX	Ti Curette	Ti Curette + H_2O_2	Ti Curette+ CHX
**Control**	-									
**1.Laser**	1.000	-								
**2.Laser + H_2O_2**	1.000	1.000	-							
**3.Laser + CHX**	1.000	1.000	1.000	-						
**4.Ti Brush**	1.000	1.000	1.000	1.000	-					
**5.Ti Brush+ H_2O_2**	1.000	1.000	1.000	1.000		-				
**6.Ti Brush + CHX**	0.075	0.362	1.000	1.000	1.000	1.000	-			
**7.Ti Curette**	0.002**	0.016*	0.276	0.169	1.000	1.000	1.000	-		
**8.Ti Curette + H_2O_2**	0.001**	0.005**	0.105	0.062	1.000	1.000	1.000	1.000	-	
**9. Ti Curette + CHX**	0.045*	0.232	1.000	1.000	1.000	1.000	1.000	1.000	1.000	-

Table S2. Pairwise comparison of Rq between groups via post hoc analysis. Dunn Bonferroni Test *$p<0.05$ **$p<0.01$.

Rq	Control	Laser	Laser + H_2O_2	Laser + CHX	Ti Brush	Ti Brush+ H_2O_2	Ti Brush + CHX	Ti Curette	Ti Curette + H_2O_2	Ti Curette+ CHX
**Control**	-									
**1.Laser**	1.000	-								
**2.Laser + H_2O_2**	1.000	1.000	-							
**3.Laser + CHX**	1.000	1.000	1.000	-						
**4.Ti Brush**	1.000	1.000	1.000	1.000	-					
**5.Ti Brush+ H_2O_2**	1.000	1.000	1.000	1.000		-				
**6.Ti Brush + CHX**	0.072	0.267	1.000	1.000	1.000	1.000	-			
**7.Ti Curette**	0.005**	0.025*	0.224	0.146	1.000	1.000	1.000	-		
**8.Ti Curette + H_2O_2**	0.002**	0.011*	0.118	0.075	1.000	1.000	1.000	1.000	-	
**9.Ti Curette + CHX**	0.081	0.295	1.000	1.000	1.000	1.000	1.000	1.000	1.000	-

Table S3. Pairwise comparison of Rt between groups via post hoc analysis. Dunn Bonferroni Test **p*<0.05 ***p*<0.01.

Rt	Control	Laser	Laser + H_2O_2	Laser + CHX	Ti Brush	Ti Brush+ H_2O_2	Ti Brush + CHX	Ti Curette	Ti Curette + H_2O_2	Ti Curette+ CHX
**Control**	-									
**1.Laser**	1.000	-								
**2.Laser + H_2O_2**	1.000	1.000	-							
**3.Laser + CHX**	1.000	1.000	1.000	-						
**4.Ti Brush**	1.000	0.002**	0.009**	0.004**	-					
**5.Ti Brush+ H_2O_2**	1.000	1.000	1.000	1.000	1.000	-				
**6.Ti Brush + CHX**	1.000	0.004**	0.018*	0.009**	1.000	1.000	-			
**7.Ti Curette**	1.000	1.000	1.000	1.000	1.000	1.000	1.000	-		
**8.Ti Curette + H_2O_2**	0.040*	0.001**	0.006**	0.003**	1.000	1.000	1.000	1.000	-	
**9.Ti Curette + CHX**	1.000	1.000	1.000	1.000	1.000	1.000	1.000	1.000	1.000	-

Table S4. Pairwise comparison of contact angle parameters between groups via post hoc analysis. Dunn Bonferroni Test **p*<0.05 ***p*<0.01.

Contact Angle	Negative Control	Positive Control	Laser	Laser + H_2O_2	Laser + CHX	Ti Brush	Ti Brush+ H_2O_2	Ti Brush + CHX	Ti Curette	Ti Curette + H_2O_2	Ti Curette+ CHX
**Negative control**	-										
**Positive control**	1.000	-									
**1.Laser**	1.000	1.000	-								
**2.Laser + H_2O_2**	0.001**	1.000	0.848	-							
**3.Laser + CHX**	1.000	1.000	1.000	1.000	-						
**4.Ti Brush**	1.000	1.000	1.000	1.000	1.000	-					
**5.Ti Brush+ H_2O_2**	0.001**	0.136	0.006**	1.000	0.780	0.039*	-				
**6.Ti Brush + CHX**	0.001**	1.000	0.872	1.000	1.000	1.000	1.000	-			
**7.Ti Curette**	1.000	1.000	1.000	1.000	1.000	1.000	0.027*	1.000	-		
**8.Ti Curette + H_2O_2**	0.001**	0.252	0.001**	1.000	1.000	0.001**	1.000	1.000	1.000	-	
**9.Ti Curette + CHX**	0.001**	1.000	0.304	1.000	1.000	1.000	1.000	1.000	0.947	0.001**	-
